# Facioscapulohumeral muscular dystrophy type 1 combined with becker muscular dystrophy: a family case report

**DOI:** 10.3389/fgene.2024.1522203

**Published:** 2025-01-07

**Authors:** Menglin Tan, Huiyi Huo, Jieming Feng, Chandi Wang, Suhua Jiang

**Affiliations:** The First People’s Hospital of Foshan, Foshan, China

**Keywords:** FSHD1, BMD, FSHD1 combined with BMD, genetic diagnosis, case report

## Abstract

Facioscapulohumeral muscular dystrophy type 1 (FSHD1) and Becker muscular dystrophy (BMD) are distinct disorders caused by different genetic variations and exhibiting different inheritance patterns. The co-occurrence of both conditions within the same family is rare. In this case report, the proband was a 10 year-old boy who presented with eye and mouth orbicular muscles, shoulder and proximal upper and lower limbs weakness. Genetic testing showed that the number of D4Z4 repeat units in the sub-terminal region 4qA of chromosome 4q35 in the proband was only 4 (normal value ≥ 11) and, at the same time, a heterozygous deletion was found in exons 13–29 of *DMD* gene in the proband, thus the diagnosis was clinically and genetically compatible with both FSHD1 and BMD. Pedigree investigation revealed that his maternal grandmother, mother, aunt and cousin also had muscle weakness in the face, shoulders and limbs. Genetic testing confirmed that each of the four relatives had four D4Z4 repeats in the 4qA region, and all of them carried a heterozygous deletion in exons 13–29 of *DMD*. Based on the X-linked features of DMD/BMD, the maternal grandmother, mother, and aunt were diagnosed with FSHD1 combined with *DMD* deletion carriers, and the male cousin was diagnosed with FSHD1 combined with BMD. This study identifies a family with a co-occurrence of clinically overt FSHD1 and BMD, which has important reference value for the diagnosis and treatment of hereditary myopathies.

## Introduction

Facioscapulohumeral muscular dystrophy (FSHD) is an autosomal dominant muscle disease with a prevalence of 1/10,000–1/20,000 (Lunt and Harper, 1991; Deenen et al., 2014; Tihaya et al., 2023). It is characterized by progressive and asymmetric muscle weakness and atrophy affecting the face, scapular girdle, and proximal upper limbs. The disease can progress to involve the trunk, pelvic, and lower limb muscles (Pandey et al., 2012). FSHD has a highly variable clinical phenotype, ranging from asymptomatic gene carriers to individuals requiring wheelchair assistance (Lunt and Harper, 1991; Tihaya et al., 2023; Aguirre et al., 2023). FSHD comprises two types: FSHD type 1 (FSHD1) and FSHD type 2, with the majority of cases (95%) being FSHD1. Although their clinical manifestations are similar, they have different pathogeneses. FSHD1 is caused by the reduction and subsequent demethylation of the double homeobox protein 4 (DUX4) unit in the linear tandem repeat sequence D4Z4 on chromosome 4q35. This results in chromatin structure loosening and uncontrolled expression of DUX4 (Lemmers et al., 2010; Hamel and Tawil, 2018; Wijmenga et al., 1992). The D4Z4 region has two haplotypes, 4qA (with activated polyadenylate pseudo-Lamin A units) and 4qB (with inactivated pseudo-Lamin A units). Reduction in D4Z4 tandem repeat units on 4qA alone leads to FSHD1 (Lemmers et al., 2007). In the general population, the number of D4Z4 repeat units on 4qA is usually 11–150, while FSHD1 patients have only 1–10 repeats (Hamel and Tawil, 2018). The remaining 5% of FSHD cases are classified as FSHD type 2, and studies have shown that pathogenic variants in epigenetic modifiers such as *SMCHD1*, *DNMT3B*, and *LRIF1* result in hypomethylation, allowing *DUX4* expression. The number of D4Z4 repeat units in FSHD type 2 patients generally falls within the normal range (Lemmers et al., 2012; van den Boogaard et al., 2016; Hamanaka et al., 2020).

Becker muscular dystrophy (BMD) is an X-linked recessive disorder caused by dystrophin in-frame deletion in the *DMD* gene. The prevalence of BMD is approximately 1.6 per 100,000 people in the general population, with an incidence of about 5.4 per 100,000 surviving male infants (Mendell et al., 2012; Salari et al., 2022). Most female carriers exhibit a normal phenotype, while a few may show symptoms due to mechanisms such as reciprocal X chromosome/autosome translocations or uniparental diploidy (Giliberto et al., 2014; Brioschi et al., 2012). BMD is characterized by progressive and symmetric weakness and atrophy of the scapular girdle, pelvic girdle, and proximal muscles of the lower limbs. Myocardial involvement and myocardial fibrosis are also frequently features of BMD (Gegenava et al., 2022; Silva et al., 2017). Moreover, it is frequent to observe pseudohypertrophy of the calves in BMD patients, along with Gower’s sign and significantly elevated creatine kinase (CK) levels (Rudnik-Schöneborn et al., 2008).

Despite their distinct clinical features, FSHD and BMD exhibit a wide range of overlapping phenotypic spectrum, making diagnosis and differential diagnosis based solely on clinical features challenging. These diseases have different genetic patterns and the occurrence of both conditions within the same individual is extremely rare, with an incidence of FSHD combined with BMD reported only once by German researchers in 2008 (Rudnik-Schöneborn et al., 2008). In this study, we describe five individuals with FSHD1 in a Chinese lineage, two of whom also have BMD. The two patients with both FSHD1 and BMD showed similar facial, shoulder, and upper limb symptoms as the other three patients with FSHD1 combined with *DMD* deletion carriers, but also showed mild pseudohypertrophy of the calf muscles and abnormal increases in CK values that were not present in the other three patients. These cases are sporadic and pose a diagnostic challenge.

## Case presentation

### Clinical manifestations of the proband

The proband is a 10 year-old boy who presented to the Department of Pediatrics at the First People’s Hospital of Foshan in January 2022 with a chief complaint of “progressive muscle atrophy in the face, shoulders, and proximal limbs over the past 3 years.” He is the third child of his parents born at full term with an uneventful delivery and no history of birth asphyxia (the first pregnancy resulted in premature delivery and death at 28 weeks due to premature rupture of membranes, while the second pregnancy was uneventful). His developmental milestones, including motor and cognitive skills, were normal, and he achieved satisfactory academic performance. At the age of 7, he began to experience significant orbicularis oculi and oris weakness, as well as in the shoulder muscles, gradually affecting the proximal muscles of the limbs. He exhibited difficulty in whistling, pursing his lips, blowing out his cheeks, and slightly impaired speech articulation, although he could still drink through a straw, close his eyes tightly, and raise his upper limbs. He displayed an abnormal posture of protruding abdomen and tiptoeing while running. He could independently climb stairs, ride a bicycle, and play basketball but experienced fatigue easily. There were no signs of recurrent rhabdomyolysis with dark-colored urine or myoglobinuria, nor were there any observations of scoliosis or lumbar hyperlordosis. Physical examination revealed mild pseudohypertrophy of the calf muscles, negative Gower’s sign, no muscle pain, and no sensory abnormalities.

**FIGURE 1:**
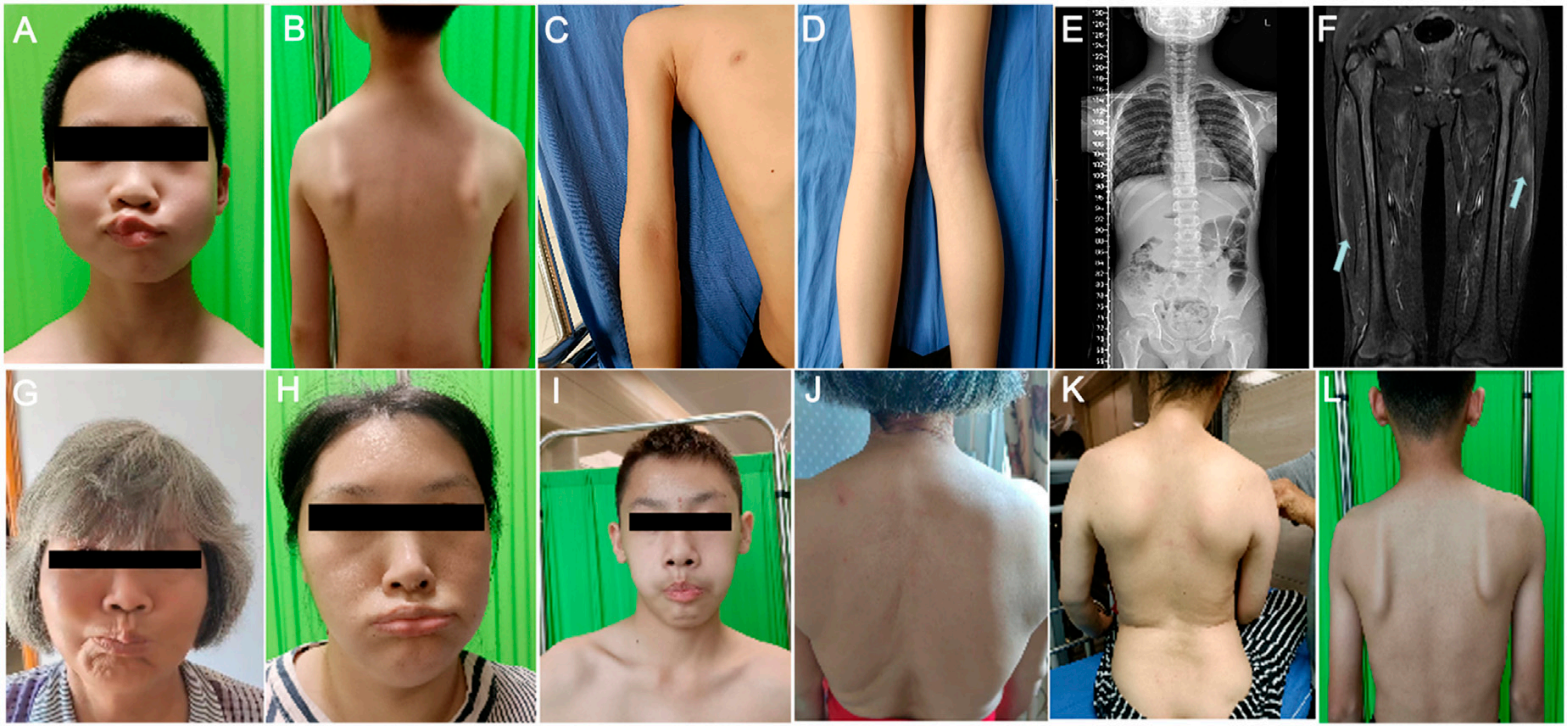
Clinical manifestations of the proband and his family members. **(A)** Facial features of the proband’s gills experiment. **(B)** Manifestations of the external shoulder blade of the proband. **(C)** Manifestations of proximal upper limb muscle atrophy in the proband. **(D)** The manifestation of proximal lower limb muscle atrophy combined with gastrocnemius pseudohypertrophy in the proband. **(E)** The entire spine of the proband shows X-ray results of mild scoliosis. **(F)** MRI of the proband’s bilateral lower limbs revealed widespread signal abnormalities in the thigh muscles, characterized by patchiness and high signal intensity in the T2WI fat suppression sequence. **(G–I)** The facial features of the proband’s maternal grandmother **(G)**, proband’s mother **(H)**, and proband’s cousin’s **(I)** bulging cheeks. **(J–L)** External shoulder blade phenotypes of proband’s maternal grandmother **(J)**, proband’s mother **(K)**, and proband’s cousin **(L)**.

Upon admission at the age of 10, He was conscious and oriented with a myopathic facial appearance characterized by reduced facial expressions, thick lips, weak cheek puffing, and left-sided cheek puffing (Figure 1A; Table 1). He displayed winged scapulae, muscle atrophy in the proximal limbs, and bilateral pseudohypertrophy of the calf muscles (Figures 1B–D). There were atrophy of the shoulder girdle muscles, including the deltoids, along with proximal limb muscle dystrophy affecting the biceps brachii, triceps brachii, and quadriceps femoris. No significant Poly-Hill sign was observed. His eyelid and lip closure, together with tongue protrusion, were moderately reduced, and his muscle strength was grade IV, including neck flexion, shoulder abduction, hip flexion, knee extension, and distal limb muscles. No macroglossia was evidenced. He exhibited flushing, erythema, and hyperkeratosis on the palmar surface of both hands and feet (Supplementary Figure 1), indicative of Nagashima-type palmoplantar keratoderma (PPKN). Deep tendon reflexes were normal, with no positive signs of Gower’s maneuver, Beevor’s sign, pathological reflexes, or meningeal irritation. X-rays of the spine revealed mild scoliosis (Figure 1E), while electromyography showed myogenic damage in the right biceps brachii and right rectus femoris muscles. MRI scans of the thighs showed multiple patchy signal abnormalities in various muscles of both thighs (e.g., sartorius, rectus femoris, vastus intermedius, vastus lateralis, adductor longus, adductor magnus, semitendinosus, and semimembranosus), characterized by asymmetry and signal heterogeneity. On T1-weighted imaging (T1WI), the abnormalities appeared isointense, while T2-weighted imaging (T2WI) revealed diffuse hyperintensity. The T2 fat-suppressed sequence (STIR sequence) provided clearer visualization, showing prominent hyperintensity. On diffusion-weighted imaging (DWI), the signals were high or slightly high (Figure 1F). Contrast-enhanced scans demonstrated mild to moderate enhancement in localized areas, with no significant enhancement elsewhere. Electrocardiography and echocardiography showed no abnormalities. His CK level at rest was elevated at 5006 IU/L (reference range: 50–310 IU/L), CK-MB was 110 ng/mL (reference range: 50–310 IU/L).

**TABLE 1:** Clinical and genetic characterization of the proband and his families.

Family member	Proband	Mother	Aunt	Cousin	Maternal grandmother
Age of consultation (years)	10	39	37	13	68
Onset age (years)	7	13	15	13	68
Number of D4Z4 repeat units	4 (4qA), 16 (4qB)	4 (4qA), 19 (4qB)	4 (4qA), 19 (4qB)	4 (4qA), 15 (4qB)	4 (4qA), 25 (4qB)
*DMD* gene deletion (exons 13–29)	Yes	Yes	Yes	Yes	Yes
Site of disease	Face, bilateral upper limbs	Face, limbs	Face, limbs	Face	Face
Onset age of upper limb symptoms (years)	10	13	15	None	None
Onset age of lower limb symptoms (years)	None	15	18	None	None
Scoliosis	+	+	+	-	+
Winged scapula	+	+	+	+	+
Pseudohypertrophy of the gastrocnemius muscle	+	-	-	+	-
Creatine kinase (U/L)	5,006	181	184	3,663	Absence
CSS score	0.5	5	4	0.5	0.5
Age-corrected CSS score	100	256	216	76	14

### Clinical manifestations of proband’s family members

In the proband’s family, there are four individuals: maternal grandmother, mother, aunt, and cousin, all exhibiting varying degrees of facial, shoulder, and/or limb muscle weakness symptoms (Figures 1G–L; Table 1). Among them, the mother and aunt of the proband had the most severe symptoms, losing the ability to walk at the ages of 30 and 37, respectively. The proband’s maternal grandmother had the mildest manifestations, with only facial and shoulder manifestations. The proband’s cousin had only facial and shoulder symptoms in terms of muscle atrophy, but he shared the proband’s false hypertrophy of the lower extremity muscles and an abnormal CK value (CK value of 3,663). In addition, the proband’s cousin also had cardiac abnormalities, manifested as sinus arrhythmia, high voltage in the left ventricle, and cardiac ultrasound indicating tricuspid valve insufficiency (mild). In terms of age of onset, the proband has the youngest age of onset, followed by the proband’s cousin, the proband’s mother and the proband’s aunt, and finally the proband’s maternal grandmother (Table 1). These results indicate that male patients differ significantly from female patients in terms of age of onset, abnormal CK values, and false hypertrophy of lower limb muscles in the proband family.

### Treatment performance of the proband

The proband started taking prednisone (30 mg/d) after diagnosis, supplemented with calcium and gastric protective medications (calcium carbonate D3 chewable tablets, once daily, one tablet per dose; aluminum magnesium carbonate tablets, three times daily, one tablet per dose). A cardiac ultrasound performed at the age of 12 revealed mild tricuspid valve regurgitation, leading to the initiation of oral captopril (18.75 mg per dose, three times a day). By the age of 13, the cardiac ultrasound showed no worsening compared to previous results, and CK level decreased to 802 U/L. There was no significant worsening in the strength of the facial and limb muscles, but Achilles tendon contracture and pseudohypertrophy of the calf muscles became more pronounced. Due to the short follow-up period, the progression of the condition will continue to be closely monitored. The patient is currently continuing medication treatment and rehabilitation training.

The proband’s cousin began taking prednisone (35 mg/d) after diagnosis, also supplemented with calcium and gastric protective medications as did the proband. At the age of 15, the child developed side effects such as hypertension and obesity, and the cardiac ultrasound indicated mild tricuspid regurgitation. Consequently, the prednisone dose was reduced to 30 mg/d, and oral captopril treatment was added (25 mg per dose, three times a day). After 18 months of treatment/follow-up, the cardiac ultrasound showed no worsening compared to previous results, and the side effects of elevated blood pressure and obesity were significantly alleviated. The CK level decreased to 1,267.8 U/L, with no significant worsening in the strength of the facial and limb muscles, and rehabilitation training continues.

The proband’s maternal grandmother, mother, and aunt did not receive any specific treatment, and their muscle weakness symptoms did not worsen during the follow-up period.

### Genetic analysis

**FIGURE 2:**
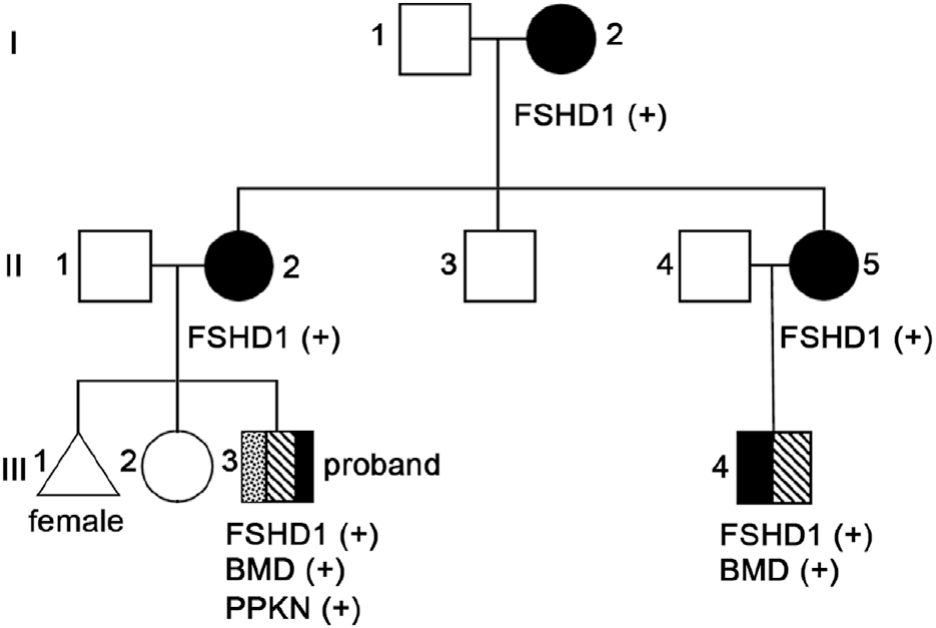
Pedigree chromatograms of the identified pathogenic variant.

Considering that the clinical manifestations of the proband and their family members also are consistent with FSHD characteristics, we conducted D4Z4 repeat length analysis and 4qA variant structure and genetic typing analysis on peripheral blood samples from the proband (III3, Figure 2), his maternal grandmother (I2, Figure 2), mother (II2, Figure 2), aunt (II5, Figure 2), uncle (II3, Figure 2) and cousin (III4, Figure 2). The results showed abnormal copy numbers of D4Z4 repeat units in the 4q35 subtelomeric region of 4qA in the proband, his maternal grandmother, mother, aunt, and cousin, while the uncle showed no abnormalities in the copy numbers of D4Z4 repeat units in the 4q35 subtelomeric region of 4qA (Table 1; Supplementary Figure 2). The proband, his maternal grandmother, mother, aunt, and cousin all had a copy number of 4 for 4qA, while the uncle had a copy number of 19 for 4qA (Supplementary Table 1). These results together with their clinical picture confirmed that the proband, his maternal grandmother, mother, aunt, and cousin were patients with FSHD1 (Figure 2).

In consideration of the atypical FSHD phenotype, a genomic exon DNA testing (performed at Shenzhen Angenomics Medical Testing Laboratory) revealed a heterozygous deletion of exons 13–29 in the proband’s *DMD* gene (Supplementary Figure 3), while qPCR verification analysis of the *DMD* gene in other family members showed that maternal grandmother, mother, maternal aunt, and cousin had a heterozygous deletion at the same site in the *DMD* gene, and no deletions were observed in the *DMD* gene exon 13–29 testing of maternal grandfather, father, and maternal uncle (Supplementary Figure 4). These results, combined with the fact that in-frame deletion in *DMD* can lead to BMD, suggest that the proband and his cousin seem to present with a BMD phenotype (Tuffery-Giraud et al., 2009). Their pathogenic mutations were inherited from their respective mothers and, ultimately, from their maternal grandmother. Overall, the proband and his cousin simultaneously suffer from FSHD1 and BMD (Figure 2), which is extremely rare. Compared with proband’s maternal grandmother, mother, and aunt, the proband and his cousin had lower extremity symptoms at the onset of the disease, especially mild pseudohypertrophy of the calf muscles, and significantly increased CK values, which are more compatible with the BMD condition (Table 1). This suggests that having both FSHD1 and BMD may further aggravate symptoms.

Furthermore, we found a compound heterozygous mutation in the *SERPINB7* gene of the proband, inherited from both parents (Supplementary Figure 5). The father carried the pathogenic nonsense variant NM_003784.3:c.796C > T (p.Arg266Ter) in the *SERPINB7* gene (OMIM603357), while the mother carried the pathogenic frameshift variant NM_003784.4:c.650_653del (p.Ser217LeufsTer7). The proband inherited both variants. According to OMIM, *SERPINB7* variants are associated with PPKN. These variants are reported pathogenic based on ClinVar annotations (https://www.ncbi.nlm.nih.gov/clinvar/). Therefore, this variation may be the cause of the proband’s PPKN. Subsequent Sanger site verification of the cousin’s *SERPINB7* gene revealed no mutations at both sites, consistent with the absence of PPKN in the cousin.

## Discussion

This paper presents a Chinese family with a co-occurrence of two different muscular dystrophies, in which the proband and four relatives exhibit varying degrees of progressive weakness and muscle weakness in the facial, shoulder girdle, and proximal upper limb muscles (Figure 2; Table 1). Molecular genetic testing revealed significantly reduced D4Z4 repeat unit numbers in 4qA alleles in all five individuals, indicating the presence of the FSHD1 genetic signature (Table 1; Supplementary Figure 2). Surprisingly, within this family, both the proband and his cousin have been further investigated considering the atypical FSHD phenotype, unveiling another concomitant genetic condition, namely, BMD from *DMD* in-frame gene deletion (Figure 2). It is extremely rare for a patient to simultaneously exhibit both FSHD1 and BMD, two different genetic forms of muscular dystrophy, with only one such case reported by German researchers in 2008. The case they reported involved a 12 year-old patient presenting with mental disorders and proximal muscle weakness affecting the shoulder girdle and facial muscles, with CK levels exceeding 7000 U/L. Genetic testing revealed a shortened D4Z4 fragment in the patient, along with a splice donor site variant (c.4071 + 1 G > T) in the *DMD* gene, confirming the diagnosis of BMD combined with FSHD (Rudnik-Schöneborn et al., 2008). The two FSHD1-BMD patients identified in this study not only manifested FSHD1 symptoms similar to the aforementioned patient, but also showed CK levels significantly exceeding five times the normal value. However, these three patients also presented with different symptoms; the case reported by German researchers showed signs of mental disorders, while the proband and their cousin in this study exhibited distinct symptoms such as pseudo-hypertrophy of the calf muscles early in the disease course, differing from FSHD1 (Table 1). Additionally, the proband’s cousin showed mild cardiac abnormalities. These findings may be attributed to the concurrent BMD diagnosis, and the differences in symptoms may be due to variations in BMD mutation sites. The patient reported by German researchers had a splice site variant (c.4071 + 1 G > T) in *DMD* exon 29 (Rudnik-Schöneborn et al., 2008), while the proband in this study had a deletion of exons 13–29 in *DMD* (Supplementary Figure 3); qPCR analysis of the proband’s cousin also revealed a deletion of *DMD* exons 13–29 (Supplementary Figure 4).

The clinical presentation of the five patients with FSHD1 in this family exhibited significant heterogeneity. The mother and aunt experienced motor impairment in both upper and lower limbs, while the proband and his cousin only showed muscle atrophy in the proximal limbs but retained good muscle strength. On the other hand, the maternal grandmother displayed facial muscle weakness solely during physical examination. These findings align with the widely reported heterogeneous clinical phenotype of FSHD1 (Hamanaka et al., 2016; Wohlgemuth et al., 2018; Klinge et al., 2006). Previous studies have demonstrated a negative correlation between the copy number of D4Z4 and the severity of FSHD1. A lower D4Z4 copy number corresponds to earlier disease onset, faster progression, and an increased likelihood of losing walking ability (Goto et al., 1995). FSHD patients with 1–3 copies of D4Z4 tend to have a more severe clinical phenotype, whereas those with 7–10 copies may exhibit a milder phenotype or remain asymptomatic carriers (Statland et al., 2015). However, emerging evidence suggests that the copy number of D4Z4 is not the sole determinant of clinical heterogeneity, as patients with the same copy number can display varying phenotypes (van Overveld et al., 2003). In our study, patients diagnosed with FSHD1 shared the same number of D4Z4 repeat units but demonstrated differences in severity and progression, indicating that the number of D4Z4 repeats alone does not account for the full range of disease phenotypes.

Furthermore, some studies suggest that the severity of FSHD1 phenotype may result from the combined effect of D4Z4 repeat number and DNA methylation level, indicating a close correlation between the clinical heterogeneity of FSHD1 and incomplete penetrance of gene loci and epigenetic modifications. Van Overveld et al. found that FSHD1 patients exhibited significantly reduced DNA methylation levels in D4Z4 repeat units, leading to derepression of DUX4 expression and influencing disease severity. Importantly, methylation levels were independent of the D4Z4 repeat number (van Overveld et al., 2003). Sacconi et al. (2013) identified the *SMCHD1* gene on chromosome 18 as a modifier gene for FSHD1, potentially influencing disease severity by affecting the methylation level of the D4Z4 repeat sequence. As DNA methylation levels of D4Z4 repeat units and *SMCHD1* gene testing were not conducted in this family line, it remains uncertain whether the correlations mentioned above exist. Further genetic analyses of this family line will be conducted to further elucidate the genotype-phenotype relationships.

Some research suggests that the clinical phenotype of male FSHD patients may be more severe than that of females, and *de novo* patients may have a more severe condition than familial inheritors (Zatz et al., 1998; Zatz et al., 1995). Some scholars also believe that the younger the age of onset, the higher the likelihood of severe myopathy and faster progression (Goselink et al., 2019). However, a 2021 nationwide single-center pediatric FSHD study in the Netherlands does not support these views (Goselink et al., 2018). In the current study, although the proband developed the disease in childhood and exhibited facial muscle weakness, muscle atrophy in the proximal limbs, winged scapula, and scoliosis, the muscle weakness symptoms were not severe, and no extramuscular manifestations were observed. The proband’s age of onset is earlier than that of the mother, but their functional ability is stronger compared to the mother’s physical condition at the same age, with relatively milder symptoms. This finding contradicts the concept suggested by previous studies, which indicate a strong correlation between age of onset and disease severity. However, the early onset of the proband may be related to the combination of FSHD1 and BMD. The German researchers reported FSHD combined with BMD patients showing symptoms of muscle hypotonia and delayed language development at the age of three, receiving pediatric care (Rudnik-Schöneborn et al., 2008).

FSHD and BMD have certain overlapping symptoms, such as shoulder girdle involvement and scapular winging, which pose some challenges in clinical diagnosis, but CK level testing can help alleviate this. In FSHD, a non-destructive muscle disease affecting the sarcolemma, serum CK levels are typically mildly to moderately elevated and do not exceed five times the normal range (Tawil and Van der Maarel, 2006). On the other hand, CK activity can be significantly higher in children with BMD, exceeding five times the normal range, and even up to 20–50 times higher in those with DMD (Giliberto et al., 2014; Juan-Mateu et al., 2015). In our study, the proband and his cousin exhibited CK levels exceeding five times the normal range and pseudohypertrophy of calves, indicating the possibility of concurrent muscle diseases. Similarly, the CK levels of FSHD and BMD combined patients reported by German researchers also significantly exceeded five times the normal value (Rudnik-Schöneborn et al., 2008). Therefore, in clinical cases of FSHD, a CK activity above five times the normal value should raise suspicion of concurrent muscle diseases.

Currently, there are no definitive treatments for FSHD and BMD, and therapeutic strategies are under development. Therapeutic strategies for FSHD focus on reducing muscular inflammation using immunomodulators, increasing muscle volume and improving muscle pathology through activation of compensatory pathways that inhibit muscle atrophy, or inhibiting DUX4 gene expression and translation using antisense oligonucleotides or gene editing techniques. Other therapeutic approaches include cell therapy, exercise rehabilitation, and antioxidant therapy (Xiao et al., 2021). Treatment for BMD primarily involves a multidisciplinary and collaborative symptomatic approach, including glucocorticoid therapy, lifelong rehabilitation exercises, management of respiratory complications, treatment of cardiac disease, orthopedic surgical interventions, and psychological counseling, aimed at prolonging survival and improving quality of life for affected individuals (Juan-Mateu et al., 2015). In our study, we observed that after prednisone treatment and rehabilitation training, the CK values of the proband and his cousin decreased significantly, but their muscle weakness symptoms progressed slowly.

Additionally, the proband was diagnosed with PPKN, an autosomal recessive disorder, due to a compound heterozygous variant in the *SERPINB7* gene inherited from both parents (Supplementary Figure 5). This diagnosis was supported by the presence of erythema and hyperkeratosis on the palm. The occurrence of three different genetic disorders with distinct inheritance patterns in the proband is a rare phenomenon requiring further investigation to determine the underlying mechanisms. In conclusion, we have, for the first time, identified three diseases with different genetic modes--FSHD, BMD, and PPKN--in the same patient. Genetic testing plays a vital role in confirming the diagnosis of patients with myopathies with atypical patterns, facilitating genetic counseling, and enabling early intervention and treatment to prevent the inheritance of these genetic diseases within the family and improve the quality of life for affected individuals.

## Data Availability

The original contributions presented in the study are included in the article/Supplementary Material, further inquiries can be directed to the corresponding author.
