# Supplementary material for: Facioscapulohumeral muscular dystrophy type 1 combined with becker muscular dystrophy: a family case report
Source: Front Genet. 2025 Jan 7;15:1522203. doi: 10.3389/fgene.2024.1522203 (PMC11747468; doi:10.3389/fgene.2024.1522203)
Supplement: Supplementary file 1 [file DataSheet1.docx]

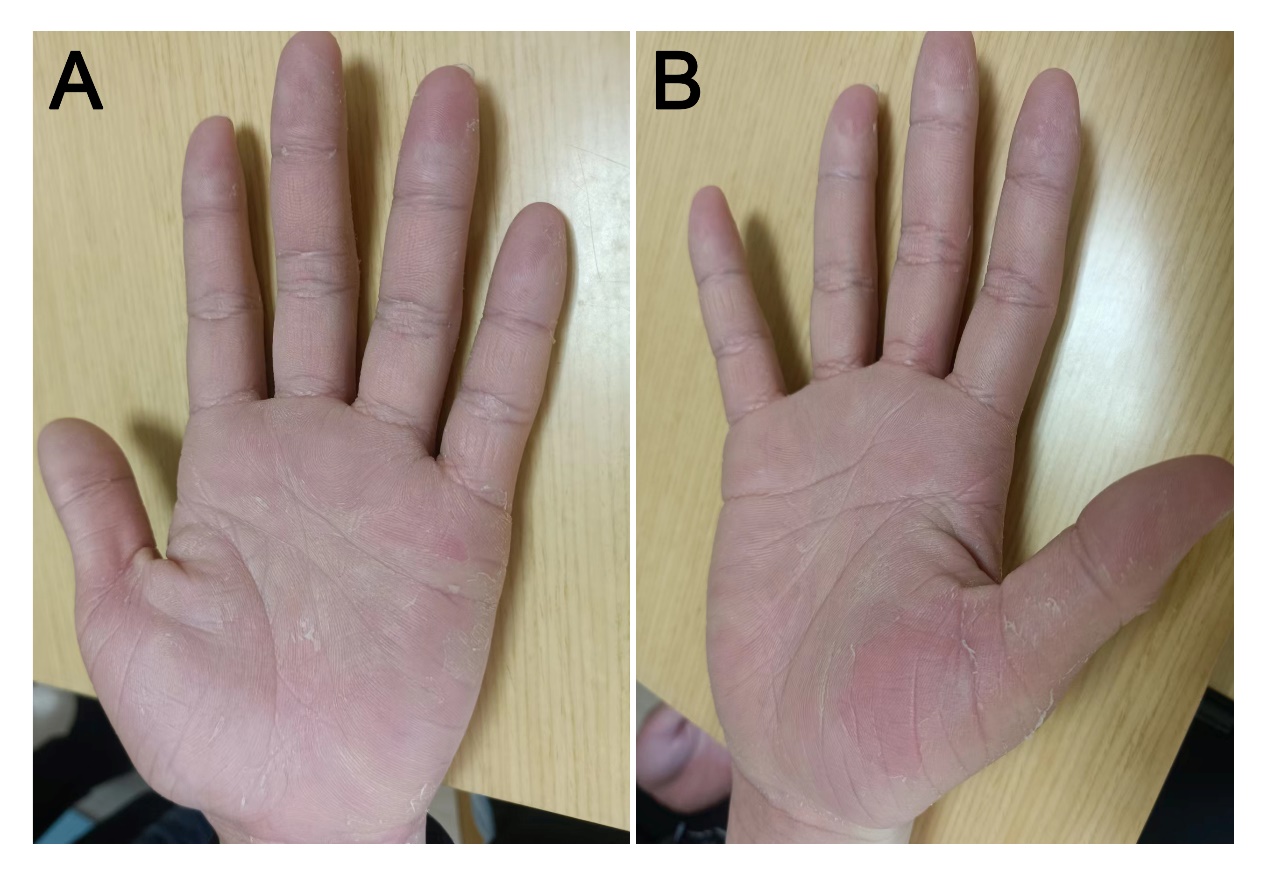


**Figure S1. The proband exhibited flushing, erythema, and hyperkeratosis on the palmar surface of hands.** (**A**) Left hand. (**B**) Right hand.


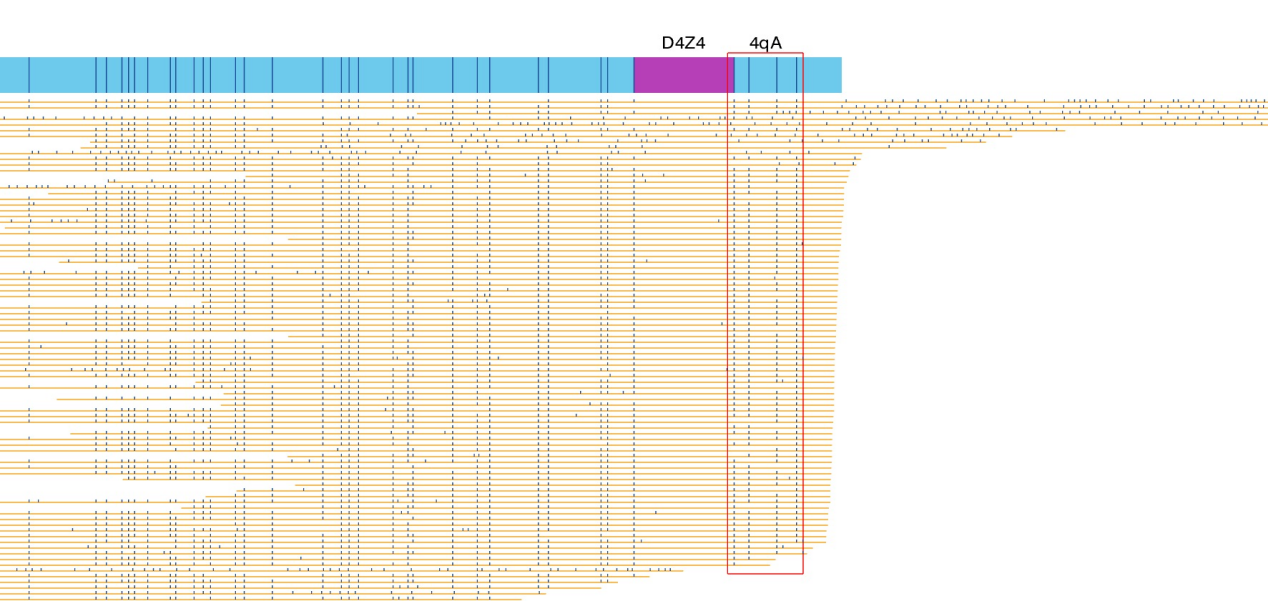


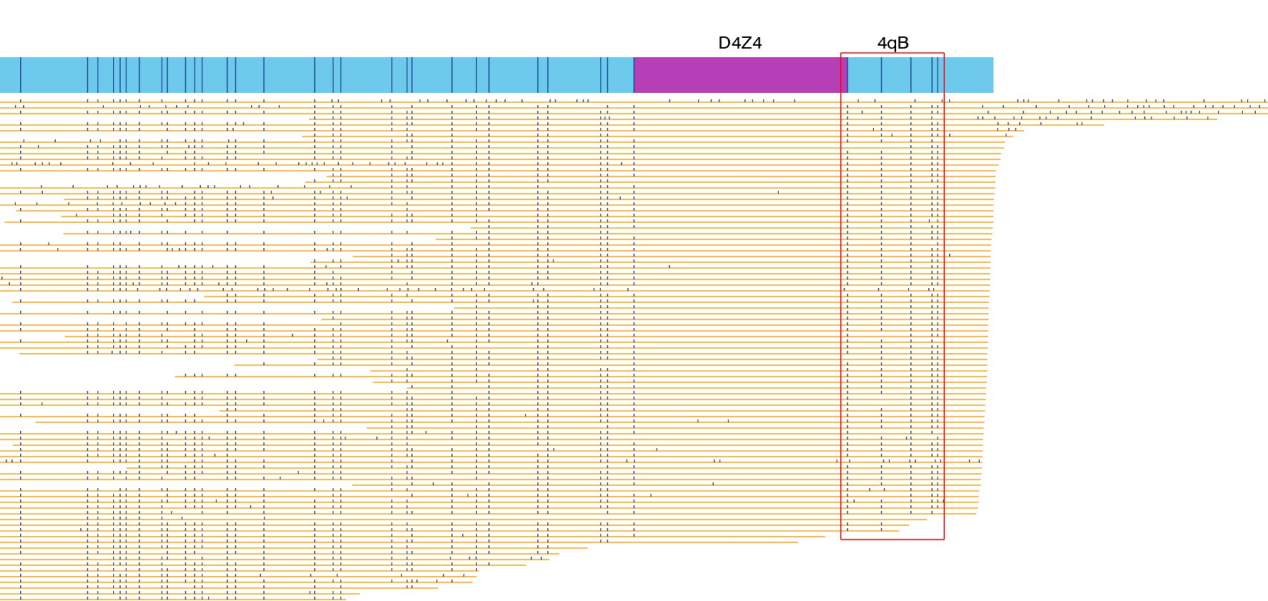


**Figure S2. The application of the Molecular Combing technique determined that the proband had 4 D4Z4 repeat units in 4qA and 16 D4Z4 repeat units in 4qB.**


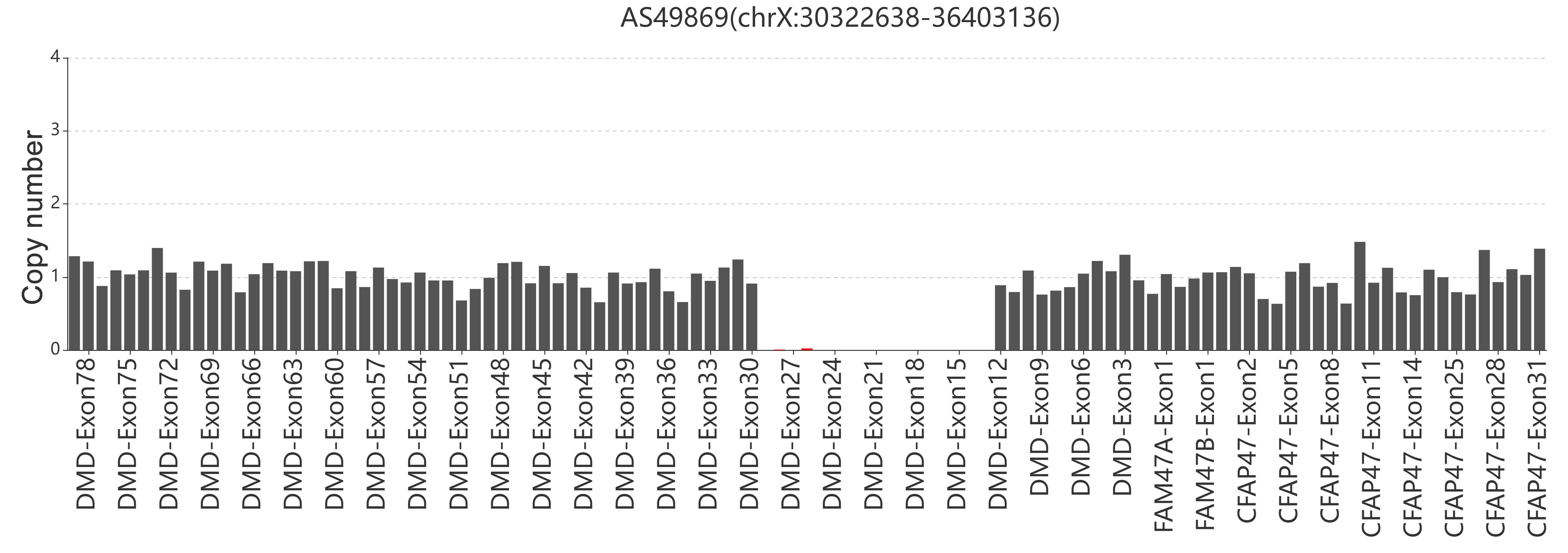


**Figure S3. *DMD* gene analysis of the proband.** Whole exon sequencing revealed deletion of *DMD* gene in proband.

**Figure S4. The DMD gene was validated in the family members of the proband.** The MightyAmp for Real-Time (TB Green Plus) qPCR reagent from Takara was used, with a relative copy number of 2 for healthy females and 1 for healthy males serving as the control group. Based on relative quantification by qPCR, the target gene copy number in the sample under investigation can be calculated by using the ΔCt value between the sample and the normal control sample, and then multiplied by the corresponding reference gene copy number.


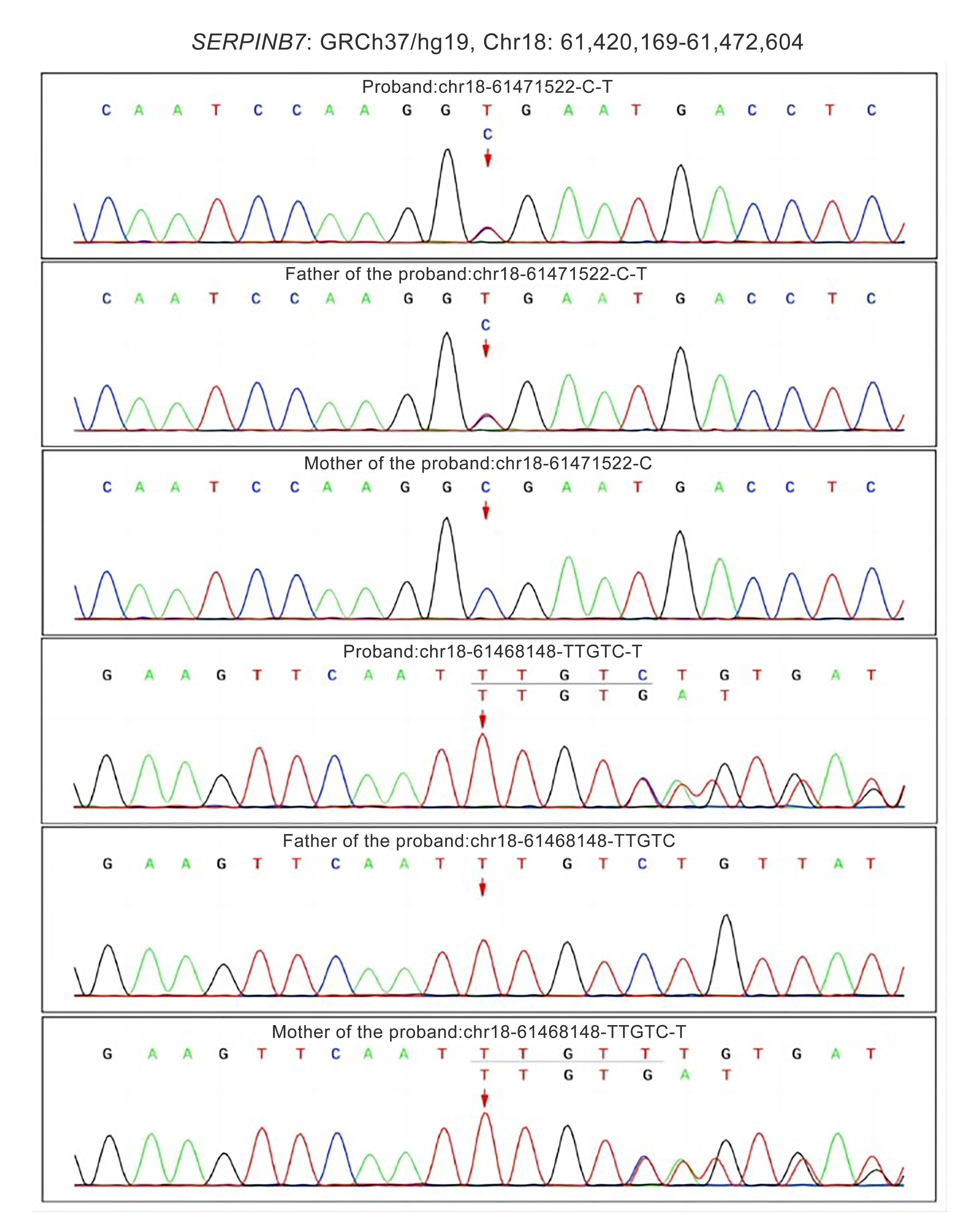


**Figure S5. The proband had compound heterozygous mutations, specifically NM_003784.3:c.796C>T (p.Arg266Ter) and NM_003784.4:c.650_653del (p.Ser217LeufsTer7), in the *SERPINB7* gene.** chr18-61471522 variation: Proband, C-T, heterozygous; Father of the proband, C-T, heterozygous; Mother of the proband, C. chr18-61468148 variation: Proband, TTGTC-T, heterozygous; Father of the proband, TTGTC; Mother of the proband, TTGTC-T, heterozygous.

**Table S1 D4Z4 repeat units of the proband's family**

| Patients | Number of D4Z4 repeat units | |
| --- | --- | --- |
| Proband’s grandmother | 4（4qA） | 25（4qB） |
| Proband’s mother | 4（4qA） | 19（4qB） |
| Proband’s aunt | 4（4qA） | 19（4qB） |
| Proband’s Cousin | 4（4qA） | 15（4qB） |
| Proband’s uncle | 19（4qA） | 25（4qB） |
